# Changes in the Firmicutes to Bacteriodetes ratio in the gut microbiome in individuals with anorexia nervosa following inpatient treatment: A systematic review and a case series

**DOI:** 10.1002/brb3.70014

**Published:** 2024-09-18

**Authors:** Passent Helal, Wangmingyu Xia, Puspendu Sardar, Anna Conway‐Morris, Andrew Conway‐Morris, Virginia A. Pedicord, Jaco Serfontein

**Affiliations:** ^1^ Adult Eating Disorders Service, Ward S3 Inpatients Addenbrooke's Hospital Cambridge UK; ^2^ Department of Medicine, Cambridge Institute of Therapeutic Immunology & Infectious Disease University of Cambridge Cambridge UK; ^3^ Adult Eating Disorder Service, Ward S3 Outpatients Addenbrooke's Hospital Cambridge UK; ^4^ School of Psychiatry NHS England Fulbourn Cambridge UK; ^5^ Division of Anaesthesia, Department of Medicine University of Cambridge Cambridge UK; ^6^ Division of Immunology, Department of Pathology University of Cambridge Cambridge UK; ^7^ John V Farman Intensive Care Unit Addenbrooke's Hospital Cambridge UK

**Keywords:** anorexia nervosa, inpatient treatment, microbiome

## Abstract

**Objective:**

Anorexia nervosa has the highest mortality rate among psychiatric illnesses. Current treatments remain ineffective for a large fraction of patients. This may be due to unclear mechanisms behind its development and maintenance. Studies exploring the role of the gut microbiome have revealed inconsistent evidence of dysbiosis. This article aims to investigate changes in the gut microbiome, particularly, mean differences in the Firmicutes to Bacteroidetes ratio, in adolescent and adult individuals with anorexia nervosa following inpatient treatment.

**Methods:**

Longitudinal studies investigating gut microbiome composition in inpatient populations of anorexia nervosa before and after treatment were systematically reviewed. Additionally, gut microbiome compositions were characterized in three acute anorexia nervosa inpatients early after admission and after 4–12 weeks of treatment.

**Results:**

Review results indicated an increase in the Firmicutes to Bacteroidetes ratio in individuals with anorexia nervosa after treatment. These however did not match values of their healthy counterparts. In the case‐series samples, the reverse occurred with samples taken 4 weeks after treatment. In the patient who provided an extra sample 12 weeks after treatment, similar results to the studies included in the review were observed. Furthermore, Firmicutes to Bacteroidetes ratio values in the case‐series samples were notably higher in the two patients who had chronic anorexia nervosa.

**Discussion:**

Differences in methodologies, small sample sizes, and insufficient data limited the generalizability of the outcomes of the reviewed studies. Results suggest a potentially unique microbiome signature in individuals with chronic anorexia nervosa, which may explain different outcomes in this group of patients.

## Graphical abstract

This article aims to investigate changes in mean differences in the Firmicutes to Bacteroidetes ratio in adolescent and adult individuals with anorexia nervosa following inpatient treatment in a systematic review and a case‐series of three individuals with anorexia nervosa.

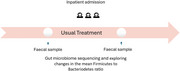

## 1 INTRODUCTION

Anorexia nervosa (AN) is a complex psychiatric disorder with unclear etiology, significant mortality rates, and medical comorbidities (Arcelus et al., 2011; Crow et al., 2009; Mehler et al., 2010; O'Brien & Vincent, 2003). At present, recommended treatments focus on weight restoration and psychological therapies despite being ineffective for the majority of patients (Frostad, 2022; Mitchell & Peterson, 2020; van den Berg et al., 2019).

In addition to the core symptoms of excessive weight loss and fear of weight gain in patients with AN, individuals often present with gastrointestinal problems in the form of reduced appetite, rapid fullness, indigestion, bloating, disordered gastric motility, constipation, and pain (Santonicola et al., 2019). Furthermore, there is evidence that the gut microbiome plays a role in modulating the gut–brain axis through different pathways, including the vagus nerve, neuroendocrine and immune systems, and influencing the behavior of the host (Fan et al., 2023; Mack et al., 2016; Rogers et al., 2016).

The human gut microbiome includes the genomes of all microorganisms inhabiting the gastrointestinal system (Aggarwal et al., 2023). However, what is most studied and referred to in this article is the bacterial microbiome. While the exact composition of a healthy microbiome is not clear, it is agreed that the genomes of certain microbial communities, known as the core microbiome, may be consistently associated with specific genotypes or environments (Aggarwal et al., 2023). In the gut, two bacterial phyla, Firmicutes and Bacteroidetes, account for around 90% of the gut microbiota, and their ratio is considered a proxy for bacterial diversity and health (Aggarwal et al., 2023; Arumugam et al., 2011; The Human Microbiome Project Consortium, 2012). This ratio, Firmicutes/Bacteroidetes, in healthy individuals from Western Europe populations appears to range from 10.3 to 1.3 (Bonder et al., 2016; Falony et al., 2016; Scepanovic et al., 2019; Turpin et al., 2016; Wang et al., 2016; Zhernakova et al., 2016).

In AN, it is hypothesized that gut dysbiosis contributes to sustained malnourishment and psychiatric symptoms via modulating signals to the hypothalamus to increase satiety, altering energy regulation as well as neurotransmitter expression and metabolism in response to food cues (Fan et al., 2023; Hildebrandt & Peyser, 2023; Nieuwdorp et al., 2014).

Several studies investigated the composition of the gut microbiome in individuals with AN before and after receiving treatment; however, results were inconsistent (Fouladi et al., 2022; Kleiman et al., 2015; Mack et al., 2016; Million & Raoult, 2018; Monteleone et al., 2021; Prochazkova et al., 2021; Roubalova et al., 2021; Schulz et al., 2021). This may have been the result of several factors such as differences in geographical location, methodologies, and the chronicity of AN in the patient population (Aggarwal et al., 2023; Allaband et al., 2019; Speciani et al., 2021). Additionally, most studies focused on healthy controls as the comparator, which may not be as useful due to known effects of diet and malnutrition on the gut microbiome. Limited data exist on a potential gut microbiome signature and relationship with the duration and chronicity of AN. Building on this, this article aims to investigate changes in the gut microbiome, particularly, mean changes in the Firmicutes to Bacteroidetes ratio (F/B) in adolescents and adult individuals with anorexia nervosa following inpatient treatment. Exploring this in cohort studies may help develop new hypotheses that can be further explored to answer etiological and prognostic questions. Hence, cohort studies with similar AN populations, interventions, comparators, and outcomes were systematically reviewed and the mean changes in F/B recorded for analysis. Additionally, changes in the gut microbiome were characterized in a case‐series comprising three individuals with anorexia nervosa admitted for inpatient treatment at a single site (S3 Ward, Addenbrooke's Hospital) in Cambridge, United Kingdom. Analysis of the gut microbiome was performed on fecal samples collected early after admission and 4–12 weeks following treatment. Primary outcomes were mean changes in F/B in AN participants from baseline. This was calculated following 16S rRNA sequencing and analysis of microbiome data.

## 2 METHODS

### 2.1 Systematic review methods

#### 2.1.1 Search strategy

**FIGURE 1:**
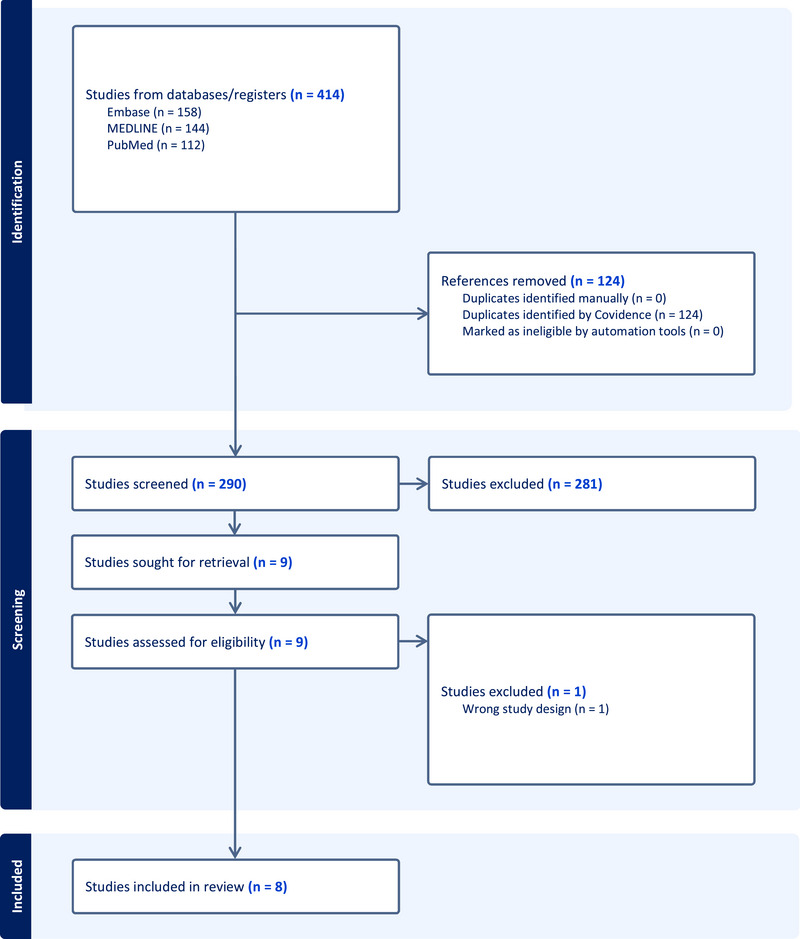
Preferred Reporting Items for Systematic Reviews and Meta‐analyses diagram for reviewed studies.

A project plan was outlined and registered on the International Prospective Register of Ongoing Systematic Reviews (Registration ID: CRD42023467242) (Booth et al., 2012). Preferred Reporting Items for Systematic Reviews and Meta‐analyses (PRISMA) guidelines were followed (Moher et al., 2009). The MEDLINE, PubMed, and EMBASE databases were reviewed up to August, 2023. Search terms used were: ((microbiota) OR (microbiome)) OR (dysbiosis)) AND (Anorexia nervosa). The search was limited to English‐language and human studies that were published in peer‐reviewed journals. Detailed search strategy is shown in Supplementary Appendix 1. Findings were imported to “Covidence” for screening (Covidence systematic review software). Eligible studies screened for extraction were based on the PICOS strategy, where participants were adults or adolescents diagnosed with AN, interventions were inpatient treatment for anorexia nervosa, comparators were primarily individuals with AN after intervention as well as a sample of healthy control group (HCs) for reference, outcomes reported changes in the abundance of taxa in the gut microbiome in AN, and study design was a longitudinal, prospective AN cohort. Case studies and studies with no available text were excluded. Figure 1 shows a PRISMA flowchart of the review search. For primary outcome measures, the relative abundance of phyla and F/B were recorded for healthy controls and for individuals with AN before and after intervention.

#### 2.1.2 Data extraction

Data extraction was done in Excel and “Covidence” with the following included: study, sample size and groups, mean body mass index (BMI), mean age, mean BMI increase in AN groups, duration in weeks between AN groups, mean F/B, mean F/B changes, duration in weeks between assessed AN samples, and duration of living with AN in months.

#### 2.1.3 Quality assessment

Risk of bias for the included studies was assessed based on the Joanna Briggs Institute Critical Appraisal Checklist for Case‐Control Studies (Table 1). Additionally, quality of the microbiome workflow was assessed by recording methodologies employed for sample collection, processing, and storage, DNA isolation, sequencing, average number of reads per sample, sequence analysis, and taxonomic assignment (Table S1).

**TABLE 1:** Literature review results.

Study	Mean F/B ratio of AN at T1	Mean F/B ratio of AN at T2	Change in F/B in AN	Average Age in years
**Kleiman 2015**	Not clear—only heatmap figure representation	Not clear—only heatmap figure representation		28
**Mack 2016**	1.22	1.42	0.2	23.8
**Monteleone 2021**	0.74	0.92	0.18	21.7
**Schulz 2021**	0.76	1.32	0.56	15.8
**Prochazkova 2021**	1.59	1.66	0.07	23
**Fouladi 2022**	1.23	1.66	0.43	25.5
**Andreani 2023**	Not reported	Not reported	Not reported	16
**Specht 2022**	Not reported	Not reported	Not reported	15.9

Abbreviations: F/B = Firmicutes/Bacteroidetes ratio, AN = anorexia nervosa, T1 = first fecal sample collected after admission, T2 = second fecal sample collected after treatment.

#### 2.1.4 Read merging and construction of abundance table and taxonomic profile

Curated fastq files were quality‐checked and processed with the Divisive Amplicon Denoising Algorithm 2 (DADA2) pipeline (Callahan et al., 2016) in R (R. R Development Core Team, 2011) v4.1.2. Briefly, we used FastQC (https://www.bioinformatics.babraham.ac.uk/projects/fastqc/) for quality checking and overall read assessment. Raw reads were checked, and Illumina universal adapters were removed using Trimmomatic v0.39 (Bolger et al., 2014). Filtered paired‐end reads were imported into DADA2 v1.16.0. An internal filtering step was performed within DADA2 allowing a maximum of two errors for both forward and reverse reads and zero “N” (undetermined nucleotide) value. After merging the paired‐end reads, a feature table containing amplicon sequence variants (ASVs) and associated abundances was generated. ASV table is a better‐resolved version of the traditional OTU table, which is produced by binning reads at a certain percentage of similarity (usually 97% for the V4 hypervariable region). After removing chimeric ASVs, sequences were classified against the Silva version 138.1 database for taxonomic assignment (Quast et al., 2013).

**FIGURE 2:**
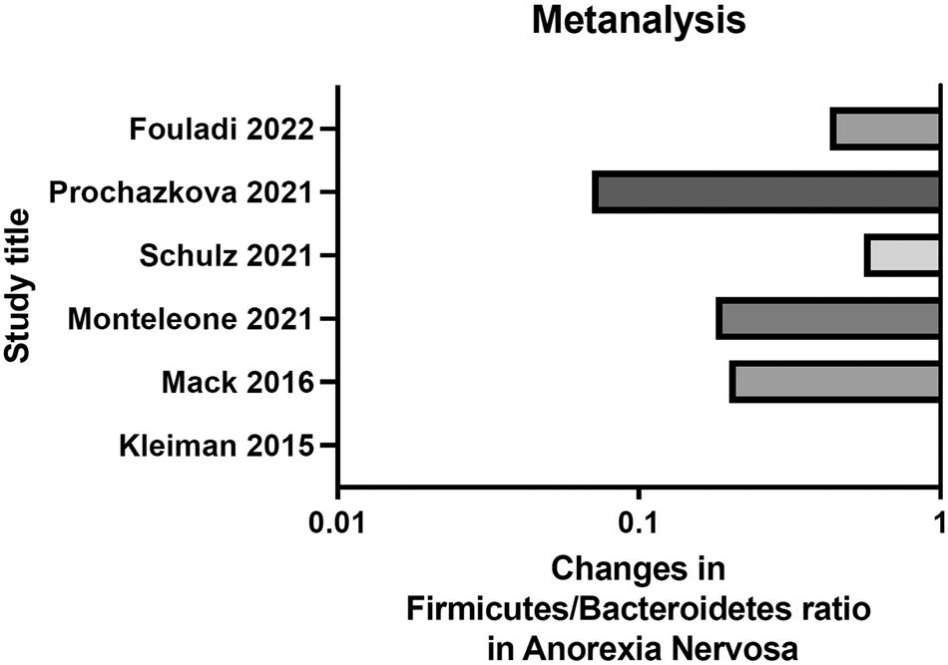
Literature review results.

Changes in mean F/B were visualized using GraphPad Prism 10 for macOS Version 10.0.2, GraphPad Software, Boston, Massachusetts USA, www.graphpad.com (Figure 2).

### 2.2 Case‐series methods

#### 2.2.1 Study population

Consenting females (*n* = 3), aged 18–65, diagnosed with AN according to the diagnostic criteria of the 10th iteration of the International Classification of Disease, and admitted to the Specialist Eating Disorder Unit (Ward S3) at Addenbrooke's Hospital, Cambridge University Hospitals, Cambridge, UK, for treatment of AN between September and December of 2022, participated in the case‐series. Exclusion criteria included possible factors that can influence the gut microbiome such as patients receiving treatment with antibiotics or pre/probiotics, those with a history of gastrointestinal tract surgery (other than appendectomy or cholecystectomy), inflammatory bowel disorder or any other diagnosis that could explain chronic or recurring bowel symptoms, or those with history of recent travel in the month prior to participating in the research.

#### 2.2.2 Assessments and treatments

Heights and weights were measured as part of the standard ward protocol. Participants were weighed upon admission and subsequently twice a week before breakfast. Participants were reminded to empty their bladders prior to weigh‐ins and were weighed in a light vest, underwear, and no shoes.

The severity of eating disorder psychopathology was measured via the Eating Disorder Examination Questionnaire (EDE‐Q) The EDE‐Q is a 28‐item measure (© 2008 by Christopher G. Fairburn and Sarah Beglin) derived from the EDE (Fairburn et al., 1993). EDE‐Q was administered on admission and after collection of the second fecal sample.

Evidence‐based therapy was delivered according to the NICE guidelines (Eating disorders: recognition and treatment [Internet], 2017). This included liaising with the multidisciplinary team for nutritional, medical, and psychological support.

Energy intake in the form of a prescribed meal plan was provided by the ward registered dietician and following the local refeeding policy, which considers each patient's previous intake, refeeding risks, and targeted weight trajectory.

Psychiatric medical reviews were conducted at least weekly with patients. Psychotropic medications were not routinely prescribed, however, patients already on psychiatric medications for comorbid conditions were continued on them.

Psychological support in the form of group “Maudsley Model of Anorexia Nervosa Treatment for Adolescents and Young Adults” was provided on the ward once weekly.

Additionally, participants received input from nursing, occupational and physiotherapists as part of the ward program and in line with the ward accreditation guidance.

#### 2.2.3 Sample collection and storage

Fecal samples were collected by participants following admission and 4 weeks and/or 12 weeks after. Following collection, samples were stored at −80°C at the Jeffrey Cheah Biomedical Centre, Cambridge, United Kingdom for subsequent DNA extraction.

#### 2.2.4 DNA extraction, 16S rRNA sequencing, and analysis

DNA extraction was performed using the FastDNA SPIN kit for soil (MP Biomedicals) as per the manufacturer's instructions. Library generation was accomplished using the 16S Barcoding Kit 1–24 (Oxford Nanopore Technologies). Before sequencing, PCR amplification and DNA concentration assessment were performed using the Qubit 4 Fluorometer (ThermoFisher). The sequencing process was conducted on the MinION Mk1B nanopore sequencer using the MinION software (Oxford Nanopore Technologies). Basecalling of raw fast5 reads was achieved using the Guppy toolkit, which contains basecalling algorithms from Oxford Nanopore Technologies. Subsequently, Porechop was employed to remove sequencing adapters. The trimmed sequences' quality was assessed using NanoPlot, and then NanoFilt was used for further quality filtering. Finally, the filtered reads were aligned against the Mouse Gastrointestinal Bacteria Catalogue and Unified Human Gastrointestinal Genome v2.0.1 database MGBC and UHGG v2.0.1 database using the classifier Kraken v2.1.2 and Bracken v2.6.2 (Almeida et al., 2021; Beresford‐Jones et al., 2022; Lu et al., 2017; Wood & Salzberg, 2014).

Kraken–Bracken analysis was conducted and obtained results were imported into Pavian, an interactive browser application, where both absolute and relative abundances at each taxonomy level were retrieved. The data were then subjected to further analysis using Microsoft Excel and RStudio (Version 1.4.1103). For visualizing the gut microbiota configuration, stacked column charts were generated. To calculate alpha diversity, the Shannon and Simpson indexes were used, accomplished with the R package “vegan.” Beta diversity was assessed through Multi‐Dimensional Scaling based on Bray–Curtis Dissimilarity at the genus level, utilizing the R package “vegan.” The R packages “dplyr,” “ggplot2,” and “ggpubr” were used to generate the data and plot the results.

Changes in F/B were calculated by paired *t*‐test and visualized using performed using GraphPad Prism 10 for macOS Version 10.0.2, GraphPad Software, Boston, Massachusetts USA, www.graphpad.com.

## 3 RESULTS

### 3.1 Systematic review results

Eight studies were identified involving a total of 334 patients with AN and 333 HCs (Table 1, Figure 2). Sample sizes were relatively small ranging from 16–56 in the AN groups and 19–69 in HCs. Average BMIs were 15.44 and 17.93 in individuals with AN before and after treatment, respectively. While the average BMI in HCs was 21.20. Studies included participants from various countries including Germany, the Czech Republic, the United States, and Italy. All studies except one used 16S rRNA sequencing of fecal samples to assess the composition of the gut microbiome; however, the genomic region sequenced was not consistent (Andreani et al., 2024; Fouladi et al., 2022; Kleiman et al., 2015; Mack et al., 2016; Monteleone et al., 2021; Prochazkova et al., 2021; Schulz et al., 2021; Specht et al., 2022). All participants were females undergoing inpatient treatment intervention involving weight restoration. Only four studies reported the average duration of AN in participants, which ranged from 17.16 to 60 months (Andreani et al., 2024; Prochazkova et al., 2021; Schulz et al., 2021; Specht et al., 2022). Exclusion criteria were relatively similar excluding participants with severe physical or psychiatric illnesses, inflammatory bowel disorders, and recent antibiotics intake. The mean values of F/B were calculated in five out of the eight studies included in the review. In three studies, mean F/B were lower in individuals with AN compared to HCs (Fouladi et al., 2022; Monteleone et al., 2021; Schulz et al., 2021). The other two studies showed higher F/B in individuals with AN (Mack et al., 2016; Prochazkova et al., 2021). Other studies that did not report F/B ratios were contacted, however, not all authors were able to provide sufficient data. Interestingly, the duration of AN reported was considerably longer in the study showing higher F/B in AN versus HC groups (Prochazkova et al., 2021; Schulz et al., 2021). In all studies with recorded F/B, treatment increased this ratio to some extent. However, F/B in AN individuals never reached HCs values.

#### 3.1.1 Curation of publicly available 16S amplicon sequencing datasets

16S rRNA raw sequencing data and individual meta‐data were obtained when possible. Data were obtained through downloading from the National Library of Medicine Sequence Read Archive library or by contacting study authors.

We retrieved human metagenomic data from two publicly available datasets through the National Library of Medicine Sequence Read Archive (SRA) using the accession numbers PRJEB11199 and PRJEB38930 for Mack et al. (2016) and Prochazkova et al. (2021), respectively, using the NCBI SRA toolkit (Mack et al., 2016; Prochazkova et al., 2021). Authors for studies with no publicly available data were contacted; however, raw sequencing data and BMI metadata were available only for Prochazkova et al. (2021). Andreani et al. (2024) was still in preprint.

#### 3.1.2 Review analysis

We were unable to conclude meaningful data from the means of F/B changes in AN groups for the studies given the lack of homogeneity and sufficient data.

**FIGURE 3:**
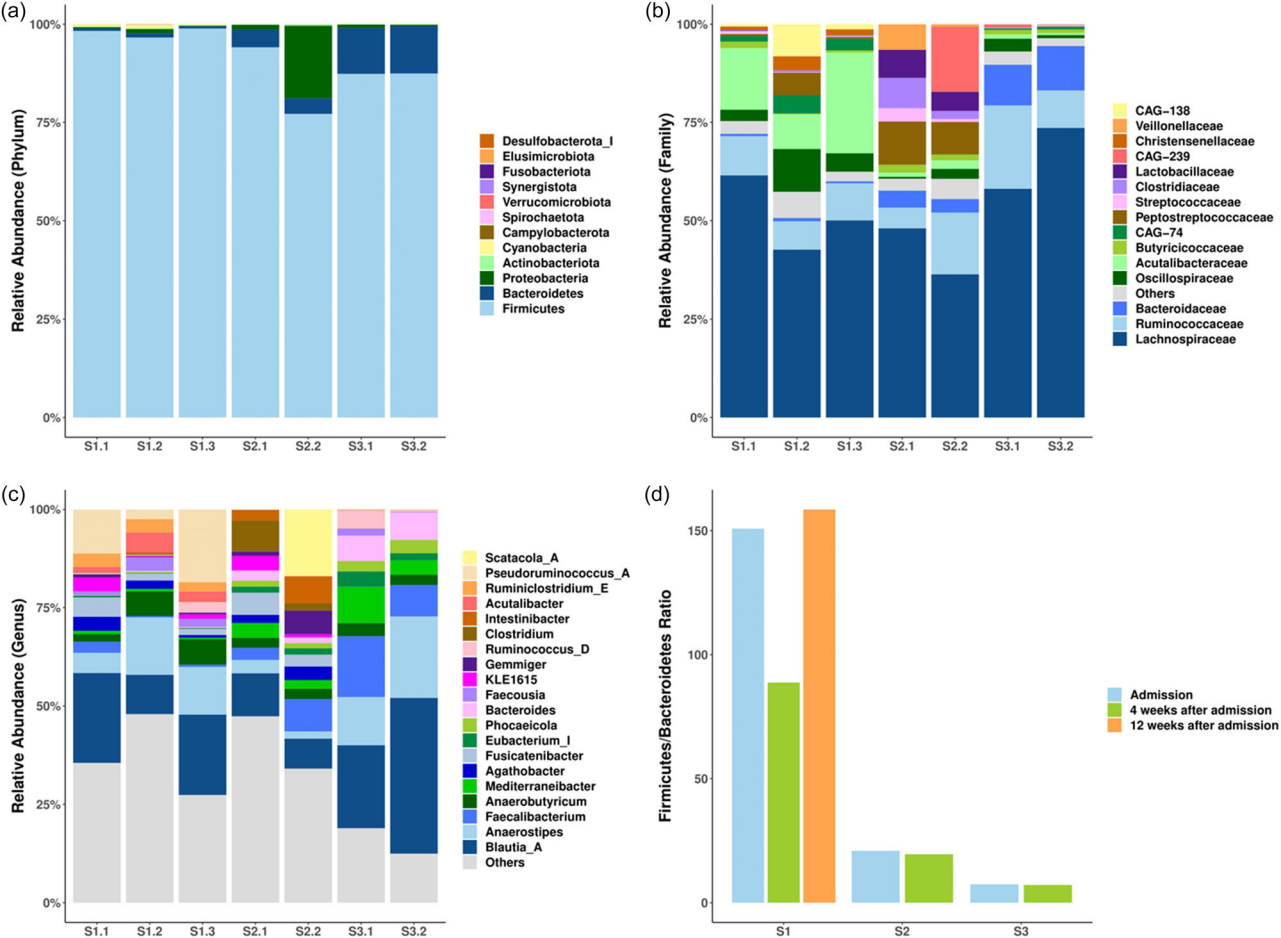
Linear regression results.

To determine if there was a relationship between F/B ratios (dependent variable) and the following parameters in individuals with AN: changes in BMI, days hospitalized, and duration of AN (independent variables), a linear regression was performed on re‐analyzed data from Prochazkova et al. (2021). This did not show significant correlations between the F/B ratios and changes in BMI or days hospitalized (Figure 3). Linear regression performed on the change in F/B and duration of AN in months suggests that further research may be indicated to inform the presence or absence of a correlation.

### 3.2 Case‐series results

Participants’ demographics and clinical characteristics are presented in Table 2. All participants reported no smoking, illicit drugs, or alcohol intake. Prior to the first and second fecal samples, all participants received a monitored food intake comprising a total energy of 1800–2200 kcal and 2300–2900 kcal, respectively.

**TABLE 2:** Case‐series demographics.

Patient	Age group	Type of AN	Years with AN	Physical comorbidities	Psychiatric comorbidities	Medications	Exercise pretreatment
1	25–30	Restricting	10	Osteoporosis	Depression	SSRIs	Excessive walking
2	50–55	Restricting	35	Osteoporosis	Depression, dependent personality disorder, self‐harm	SSRIs, anxiolytics, laxatives	Excessive walking
3	<20	Restricting and purging	2	None noted	Anxiety, depression, self‐harm	SSRIs	Excessive walking

Abbreviations: AN = anorexia nervosa, SSRIs = selective serotonin re‐uptake inhibitors.

**FIGURE 4:**
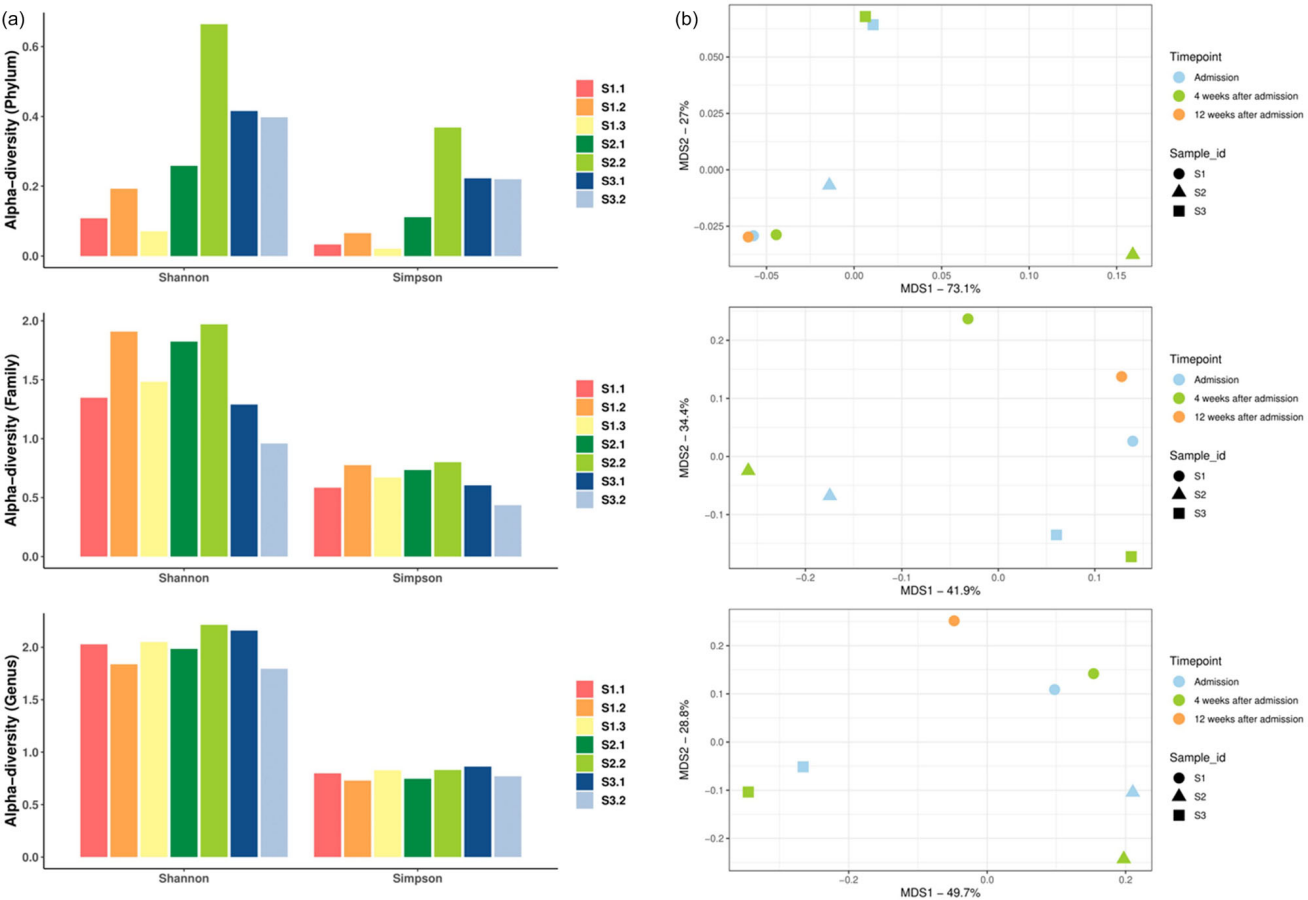
Case‐series results: changes in the composition of the gut microbiome. (a) Stacked bar chart showing the gut microbial composition of three patients at different time points at the Phylum level. (b) Stacked bar chart showing the top 15 gut microbiota with the highest relative abundances at the Family level. (c) Stacked bar chart showing the top 20 gut microbiota with the highest relative abundances at the Genus level. (d) The bar chart illustrating the Firmicutes/Bacteroidetes (F/B) ratio of three patients at different time points. For D, Wilcoxon test was used to analyze the statistical difference between sampling time points; difference between patients was compared by Mann–Whitney test, n.s., *p* > .05.

An imbalance of gut microbiota was reported in all participants, with Firmicutes being highly dominant, while Bacteroidetes and other commensal phyla showed the opposite trend (Figure 4A, Table 3). This did not notably change during treatment despite weight gain leading to a 1.1 to 1.2 increase in BMI as well as psychological improvement. In all participants, F/B decreased when comparing the first sample with that taken 4 weeks after treatment (Figure 4D). In the participant who provided an extra sample 12 weeks after treatment, F/B increased again to a level higher than the initial sample (Figure 4D). Furthermore, F/B values were higher in the two patients who have been living with anorexia nervosa for over 10 years. More specifically, F/B were notably higher in one patient and higher than those included in the review in the other (Table 3, Figure 4D).

**TABLE 3:** Case‐series results.

Sample	Patient	Timepoint	BMI	Change in BMI at 4 weeks	F/B	F/B changes	F/B changes	EDEQ	Change in EDEQ
						Between Samples 1 and 2	Samples 1 and 3		
S1.1	1	Admission^a^	12.5	1.2	150.79	−62.04	7.85	4.63	−1.73
S1.2		4 weeks after admission	13.7		88.75			2.9	
S1.3		12 weeks after admission	15.2		158.64			2.98	
S2.1	2	Admission	12.3	1.1	20.94	−1.39	n/a	6	−0.6
S2.2		4 weeks after admission	13.4		19.55			5.4	
S3.1	3	Admission	15.5	1.1	7.42	−0.26	n/a	5.46	−2.98
S3.2		4 weeks after admission	16.6		7.16			2.48	

Abbreviations: BMI = body mass index, F/B = Firmicutes to Bacteroidetes ratio, EDEQ = the Eating Disorder Examination Questionnaire.

^a^ Admission = samples taken as soon as feasible following admission (This was after 9, 7, and 21 days for patients 1, 2, and 3, respectively).

**FIGURE 5:**
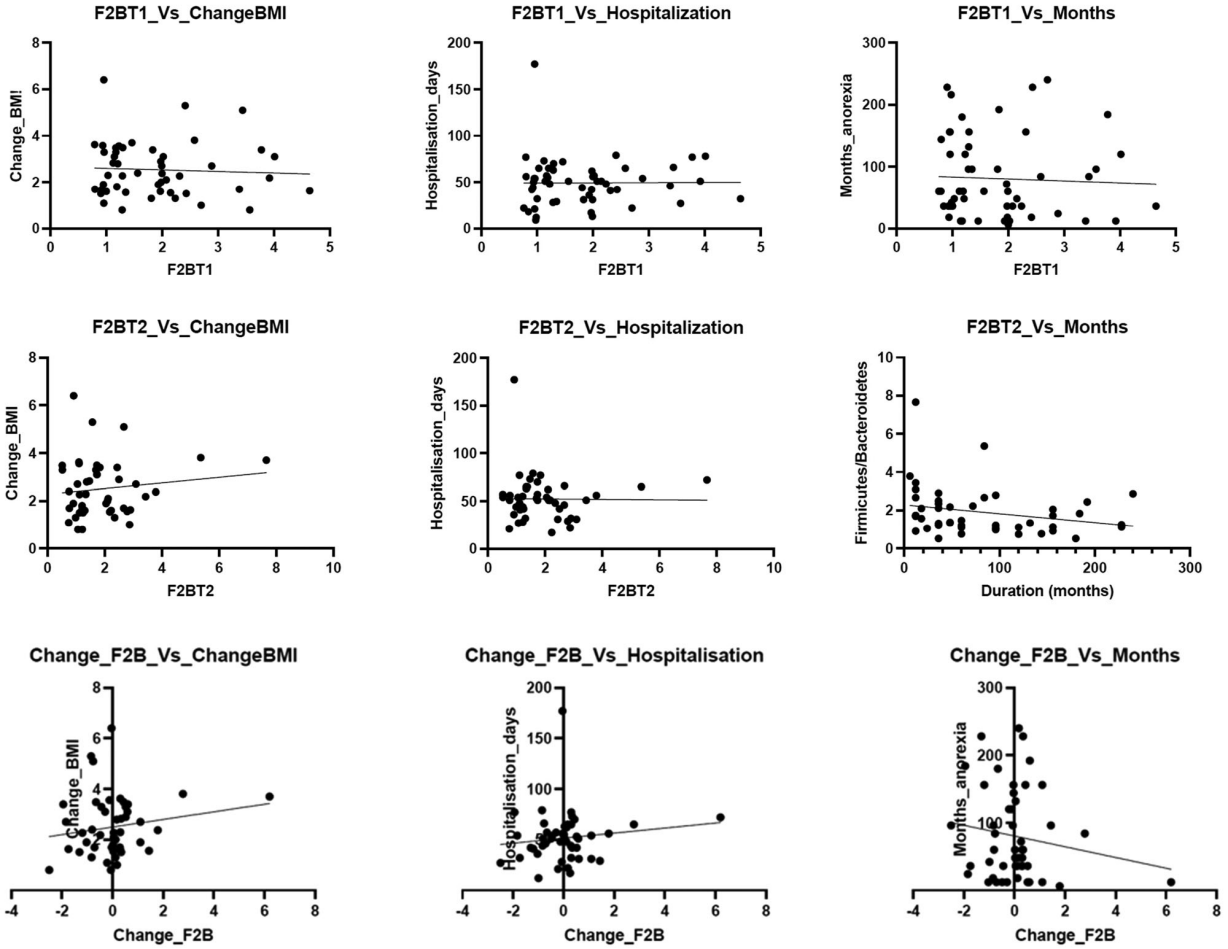
Case‐series results. (a) Bar plots illustrating the Alpha‐diversity indices (Shannon index and Simpson index) in gut microbiomes of three patients at different sampling time points at the Phylum level (top), Family level (middle), and Genus level (bottom). Wilcoxon test was used to analyze the statistical difference between sampling time points; difference between patients was compared by Mann–Whitney test. ns: *p* > .05. (b) Multiple dimension scale (MDS) plots showing the composition differences (Bray–Curtis distances) of three patients at different time points at the Phylum level (top), Family level (middle), and Genus level (bottom). PERMANOVA analysis was conducted to compare the beta diversity at three taxonomic levels.

The relative abundance of Proteobacteria was elevated in one participant 4 weeks after admission. Correspondingly, the proportion of *CAG‐239* at the family level and *Scatacola_A* at the genus level were increased in the same participant (Figure 4C). No significant changes were noted in alpha diversity measures during treatment (Figure 5A). Similarly, no significant changes were noted in beta diversity between the samples (Figure 5B).

## 4 DISCUSSION

Homeostasis of the gut microbiome is believed to be essential for maintaining a healthy intestinal structure and contributing to immune and metabolic functions (Cryan et al., 2019; The Human Microbiome Project Consortium, 2012). In addition, evidence of interactions between the gut microbiome and the brain via various pathways indicate a possible link to psychiatric symptoms and AN (Carbone et al., 2021; Fan et al., 2023; Hildebrandt & Peyser, 2023). While the exact mechanism connecting the gut microbiome with the development and maintenance of AN remains unclear, it is hypothesized that a higher F/B in individuals with AN may be contributing to its maintenance via the role of Firmicutes in increasing leptin levels and subsequently reducing appetite and fat storage (Aguirre & Venema, 2015; Magne et al., 2020). In individuals with BMIs in the obese range, evidence of higher F/B was explained by the possible role of Firmicutes as better energy extractors leading to weight gain when in higher proportion to Bacteroidetes (Krajmalnik‐Brown et al., 2012; Magne et al., 2020). Together, these findings and hypotheses demonstrate that an imbalance in the composition of the gut microbiota may promote or contribute to the maintenance of unhealthy states. This article evaluated gut microbial composition in individuals with AN before and after treatment to try to find possible patterns that may be explored in larger studies to further our understanding of AN.

The studies included in our literature review and case‐series show that the gut microbiome composition differs at different stages of AN. The case‐series results agreed with those from the literature review with regard to differences in the microbiome composition and F/B in individuals with AN following treatment and with other reviews (Di Lodovico et al., 2021). However, the direction of change in the reviewed studies was positive leading to an increase in the F/B. Whereas in our case‐series, this change was negative in the samples taken 4 weeks after treatment. In the sample taken from one patient 12 weeks after treatment, the direction of change in F/B followed the pattern seen in the reviewed studies. Although it is difficult to interpret with a small number of participants, it is recommended to analyze changes in the future from multiple, serial samples from HC and AN cohorts to explore whether these changes are transient and to have a better insight into microbiome dynamics (Allaband et al., 2019). Furthermore, since medications are believed to impact the gut microbiome, researching the effects of various medications, doses, and duration on F/B is warranted (Nikolova et al., 2021; Tomizawa et al., 2021).

Our case‐series revealed markedly high F/B in AN compared to reviewed studies. In healthy controls, F/B ranged from 10.3 to 1.3 (Bonder et al., 2016; Falony et al., 2016; Scepanovic et al., 2019; Turpin et al., 2016; Wang et al., 2016; Zhernakova et al., 2016), which is much lower than results noted from our case‐series. This contrast seemed to be more pronounced in the two patients who have been living with AN for over a decade. Interestingly, only two of the six reviewed studies reported the duration of AN in participants (Prochazkova et al., 2021; Schulz et al., 2021). In agreement with our results in participants with chronic AN (defined here as a duration of illness over 3 years years), F/B was higher in AN compared to HCs in those who had AN for an average duration of 60 months (Broomfield et al., 2017; Hay & Touyz, 2018; Prochazkova et al., 2021; Speciani et al., 2021). This further supports the hypothesis that chronic AN may have a distinct microbiome signature, which may explain its poor response to conventional treatments (Mack et al., 2016; Schulz et al., 2021).

In reviewed studies, individuals with AN did not seem to restore F/B back to HC values. This may be related to several factors such as duration of treatment received prior to sample collection but also possibly the chronicity of AN, raising questions on whether classification of AN according to duration of illness may offer new venues to explore with regard to treatment (David et al., 2014; Dhopatkar et al., 2023; Mack et al., 2016; Schulz et al., 2021; Wu et al., 2011). Furthermore, gathering functional data and integrating disease domains may provide more insight on differences between the various stages of AN and reveal any specific correlations (Aggarwal et al., 2023; Chen et al., 2019; Fouladi et al., 2022; Insel et al., 2010; Nikolova et al., 2021; Prochazkova et al., 2021).

### 4.1 Strengths and limitations

This review included studies with longitudinal AN cohorts for testing before and after treatment and a cross‐sectional HCs cohort for reference. While the prospective design was able to show valuable data in response to treatment, a cross‐over design with participants with AN and HCs undertaking the intervention and including a washout period and a long follow‐up may have controlled for intra‐ and intersubject variability and provided more insight (Johnson et al., 2020; Korem et al., 2017; Zhu et al., 2020). Additionally, obtaining multiple samples from each participant may have controlled for within‐subject variation (Johnson et al., 2020).

A major limitation to this review was the lack of openly available data and meta‐data for most of the studies. Despite our efforts to contact authors, we were not always successful. Other limitations included the differences in the intervals of collection of the second fecal sample, DNA kits, 16S rRNA primers, sequencing approaches, and data libraries used for taxonomic assignment. All these factors made a broader and higher powered meta‐analysis impossible (Allaband et al., 2019; Goodrich et al., 2014; Kim et al., 2017; Parada et al., 2016; Salter et al., 2014; Smith et al., 2011; Wu et al., 2010).

Statistical approaches adopted by the reviewed studies to analyze taxa abundance in AN patients before and after treatment included mostly nonparametric tests such as two‐tailed Mann–Whitney and Wilcoxon's tests (Kleiman et al., 2015; Mack et al., 2016; Prochazkova et al., 2021; Schulz et al., 2021). However, independent variables tested were not always clearly reported. While nonparametric models are recommended to reduce type 1 errors, permutation models are also advised given their greater power, adjustability to data clustering, and ease of use (Kleine Bardenhorst et al., 2021; Shankar, 2017; Thorsen et al., 2016). Additionally, it was noted that power calculations for effect sizes were often not clearly recorded and hence studies may have been underpowered to detect small effect sizes (David et al., 2014; Debelius et al., 2016; Sullivan & Feinn, 2012; Wu et al., 2011). Reports found that 400–500 samples per group may be needed to detect 5%–9% differences in taxa abundance, suggesting that increasing the number of samples may increase power and exclude within‐subject noise (Falony et al., 2016; Rothschild et al., 2018).

## 5 CONCLUSIONS AND FUTURE DIRECTIONS

The relationship between the gut microbiome in the pathophysiology of AN is clearly complex. The overarching aim of exploring this relationship is to develop novel effective tools for prognosis and treatment. Despite the recent surge in studies in this area, the results have been confusing and inconsistent. This seems to be largely due to individual studies being underpowered. Additionally, the lack of homogeneity in study design and methods makes it difficult to combine their results and draw conclusions. Due to numerous confounders potentially influencing the composition of the microbiome, large registries, and the utilization of computing technologies, advances in sequencing techniques, and integrating functional data is essential. Further research is needed to explore potential differences in the gut microbiome that are more specific to individuals with chronic AN that may aid in different treatments offered.

## AUTHOR CONTRIBUTIONS

PH wrote the research protocol, led research committee procedures for approvals, conducted, and wrote the review. PH and JS screened the studies, assessed quality, and extracted the data for the systematic review. AM supervised and guided the study design, protocol, and approvals. PH and WX performed laboratory procedures. WX wrote the results and methods sections of the case‐series, developed the figures, acquired the data, and performed the analyses using R. PS performed quality analysis and re‐analysis of studies reviewed where available. PS and VP performed linear regression analysis of studies reviewed. VP reviewed and advised laboratory procedures, data acquisition, analyses, and provided expertise on microbiome data. JS and ACM reviewed the protocol, advised, and supervised the project as the principal investigator and bursary supervisor, respectively. JS contributed to the case‐series design and supported recruitment of participants. All authors helped with editing the manuscript, interpretation of data, and agreed on the final version.

## CONFLICT OF INTEREST STATEMENT

AM reports speaking fees from Biomerieux, Fischer and Paykel, and Boston Scientific; he sits on the scientific advisory board of Cambridge Infection Diagnostics.

### PEER REVIEW

The peer review history for this article is available at https://publons.com/publon/10.1002/brb3.70014.

## Supporting information



Table S1. Assessment of risk of bias of studies using the Joanna Briggs Institute Critical Appraisal Checklist for Case‐Control Studies.

## Data Availability

Sequencing data are archived in the National Centre for Biotechnology Information under project number PRJNA1064020.
