# Correction: Correction to Changes in the Firmicutes to Bacteriodetes ratio in the gut microbiome in individuals with anorexia nervosa following inpatient treatment: A systematic review and a case series

**DOI:** 10.1002/brb3.70204

**Published:** 2025-02-18

Helal, P.,  Xia, W.,  Sardar, P.,  Conway‐Morris, A.,  Conway‐Morris, A.,  Pedicord, V. A., &  Serfontein, J. (2024). Changes in the Firmicutes to Bacteriodetes ratio in the gut microbiome in individuals with anorexia nervosa following inpatient treatment: A systematic review and a case series. *Brain and Behavior*, 14, e70014. https://doi.org/10.1002/brb3.70014

[Description of error]

• The figure legend under **Figure 4** should be moved to **Figure** 3.

• The figure legend under **Figure 5** should be moved to **Figure** 4.

• The figure legend under **Figure 3** should be moved to **Figure** 5.

We apologize for this error.

Please find the below figures with correct descriptions/legends under each as per above.

**FIGURE 3:**
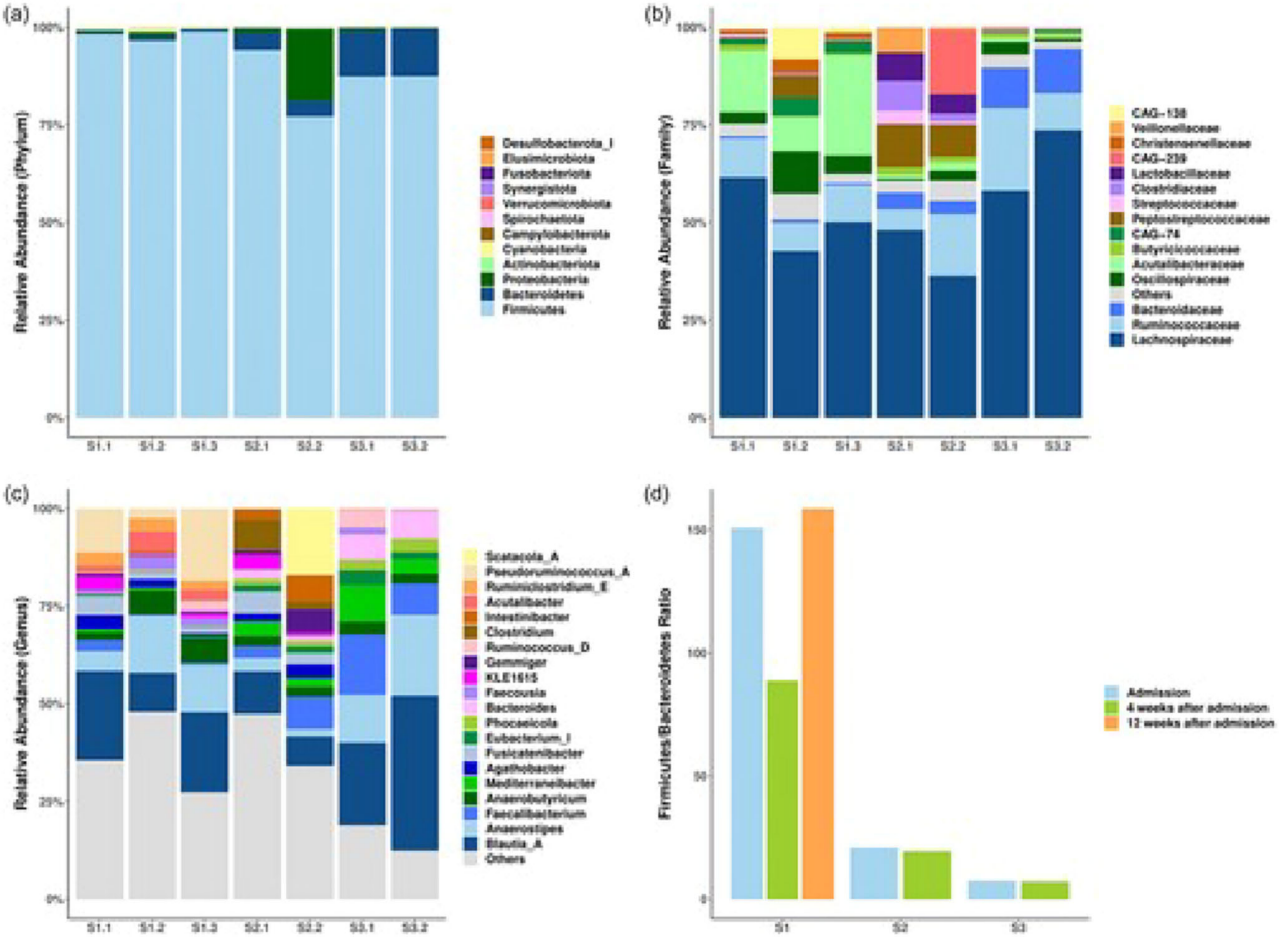
Case‐series results: changes in the composition of the gut microbiome. (a) Stacked bar chart showing the gut microbial composition of three patients at different time points at the Phylum level. (b) Stacked bar chart showing the top 15 gut microbiota with the highest relative abundances at the Family level. (c) Stacked bar chart showing the top 20 gut microbiota with the highest relative abundances at the Genus level. (d) The bar chart illustrating the Firmicutes/Bacteroidetes (F/B) ratio of three patients at different time points. For D, the Wilcoxon test was used to analyze the statistical difference between sampling time points; the difference between patients was compared by the Mann–Whitney test, n.s., *p* > 0.05.

**FIGURE 4:**
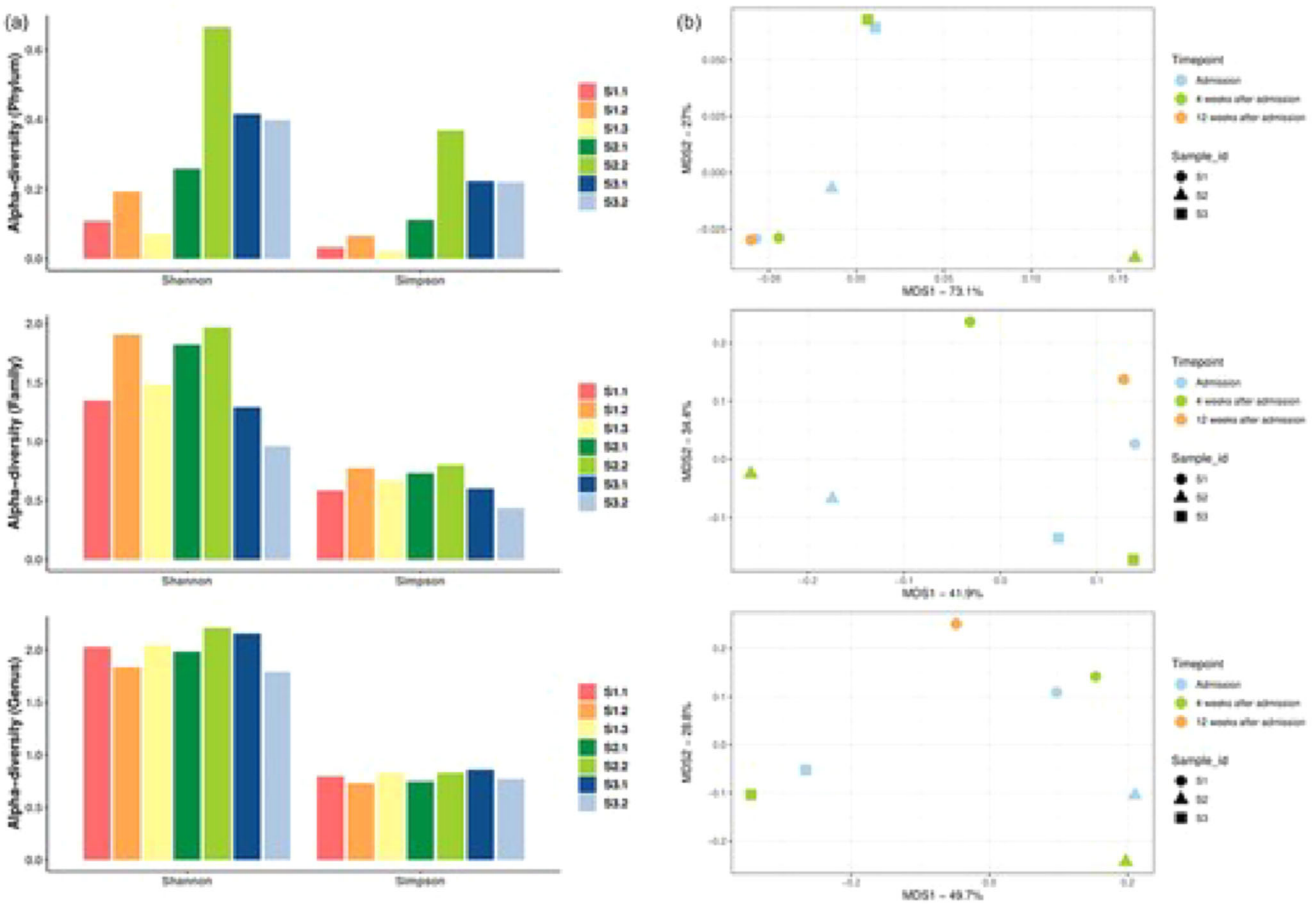
Case‐series results. (a) Bar plots illustrating the Alpha‐diversity indices (Shannon index and Simpson index) in gut microbiomes of three patients at different sampling time points at the Phylum level (top), Family level (middle), and Genus level (bottom). Wilcoxon test was used to analyze the statistical difference between sampling time points; the difference between patients was compared by the Mann–Whitney test. ns: *p* > 0.05. (b) Multiple dimension scale (MDS) plots showing the composition differences (Bray–Curtis distances) of three patients at different time points at the Phylum level (top), Family level (middle), and Genus level (bottom). PERMANOVA analysis was conducted to compare the beta diversity at three taxonomic levels.

**FIGURE 5:**
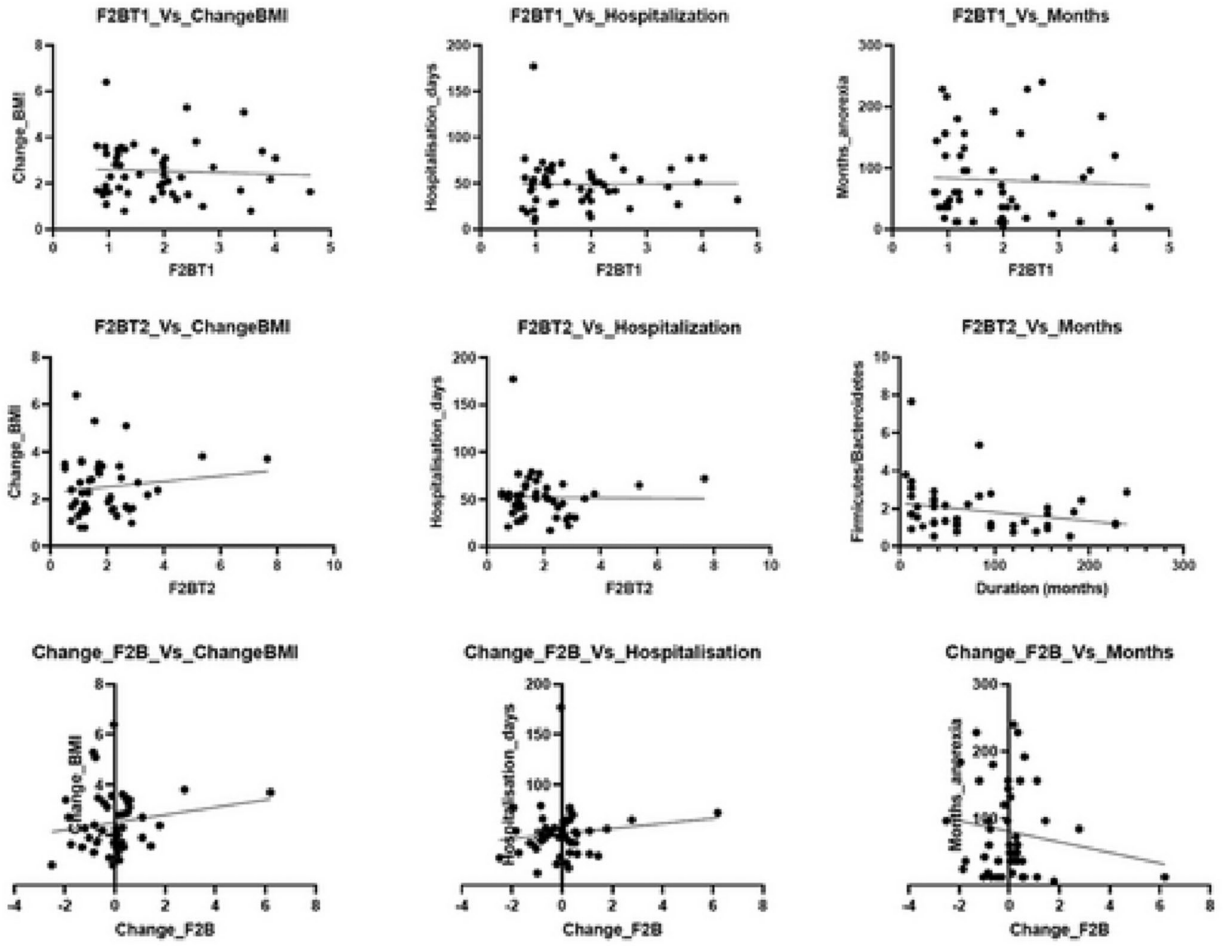
Linear regression results.

