# Supplementary material for: Changes in the Firmicutes to Bacteriodetes ratio in the gut microbiome in individuals with anorexia nervosa following inpatient treatment: A systematic review and a case series
Source: Brain Behav. 2024 Sep 18;14(9):e70014. doi: 10.1002/brb3.70014 (PMC11410858; doi:10.1002/brb3.70014)
Supplement: Supplementary file 1 — Table S1. Assessment of risk of bias of studies using the Joanna Briggs Institute Critical Appraisal Checklist for Case‐Control Studies. [file BRB3-14-e70014-s001.docx]

**Supplementary materials**

Supplementary Table 1.

*Assessment of risk of bias of studies using the Joanna Briggs Institute Critical Appraisal Checklist for Case-Control Studies*

| **Study ID** | **Were criteria for inclusion in sample clearly defined?** | **Were study subjects and setting described in detail?** | **Was exposure measured in valid and reliable way?** | **Were objective standard criteria used for measurement of the condition?** | **Were confounding factors identified?** | **Were strategies to deal with the confounding factors stated?** | **Were the outcomes measured in a valid and reliable way?** | **Were appropriate statistical analyses used?** |
| --- | --- | --- | --- | --- | --- | --- | --- | --- |
| **Kleiman 2015** | Yes | Yes | Yes | Yes | Yes | Yes | Yes | Yes |
| **Mack 2016** | Yes | Yes | Yes | Not clear | Yes | Yes | Yes | Yes |
| **Monteleone 2021** | Yes | Yes | Yes | Yes | Yes | Yes | Yes | Yes |
| **Schulz 2021** | Yes | Yes | Yes | Yes | Yes | Yes | Yes | Yes |
| **Prochazkova 2021** | Yes | Yes | Yes | Yes | Yes | Yes | Yes | Yes |
| **Fouladi 2022** | Yes | Yes | Yes | Yes | Yes | Yes | Yes | Yes |
| **Andreani 2023** | Yes | Yes | Yes | Yes | Yes | Yes | Yes | Yes |
| **Specht 2022** | Yes | Yes | Yes | Yes | Yes | Yes | Yes | Yes |
